# Supplementary material for: Mechanisms of mistrust: A Bayesian account of misinformation learning
Source: PLoS Comput Biol. 2025 May 14;21(5):e1012814. doi: 10.1371/journal.pcbi.1012814 (PMC12077715; doi:10.1371/journal.pcbi.1012814)
Supplement: S1 Text — (PDF) [file pcbi.1012814.s001.pdf]

Supplementary Information

S1 Participants

In experiment 1, we analyzed a total of 123 participants. (58 female, 63 male, 2 "other/would rather not say"). The mean age of participants was 38.72 years (SD = 11.07). In experiment 2, we analyzed a total of 111 participants. (54 female, 55 male, 2 "other/would rather not say"). The mean age of participants was 36.77 years (SD = 13.27). We show an overview of these demographics and participants education levels in Fig. A.

We excluded participants based on two criteria in both experiments: Participants were excluded it they had incorrectly responded to more than two comprehension checks in our instructions, or if they incorrectly responded to a catch-question in the post-task questionnaires.

Participants were remunerated with a base payment and a performance based bonus computed via the quadratic scoring rule based on their confidence judgements during the main block (1) .

S2 Additional results

Distribution of confidence ratings

We provide further insights into the collected dataset by testing for differences in the confidence ratings distribution *(i)* between the experiments (full feedback and noisy conditions); *(ii)* within the experiments (fixing the feedback condition, but varying

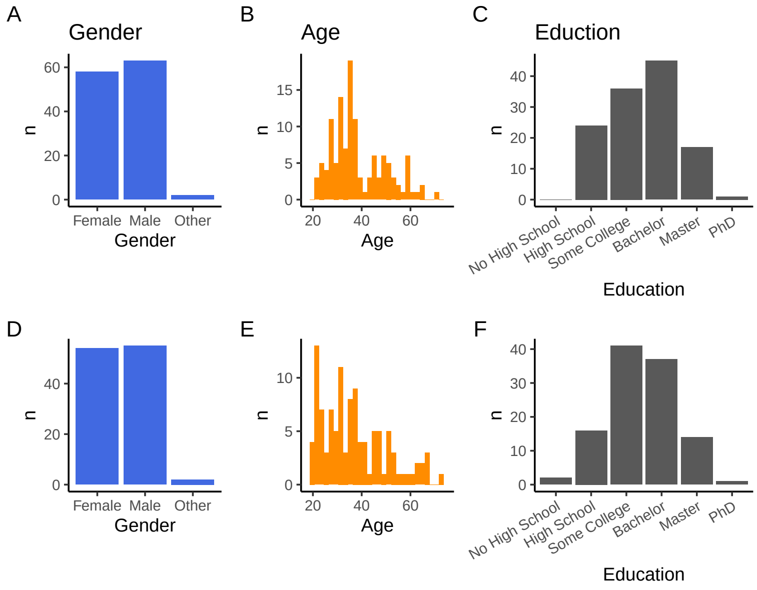

Fig A. Demographics. (A-C) Experiment 1. (D-F) Experiment 2.

Table A. Cohen's d values between different source types. We report the value pooled across full feedback and noisy, as well as the individual values (full feedback, noisy).

$\mu_1 \setminus \mu_2$	**Helpful**	**Random**	**Opposite**	**Blue-bias**
**Helpful**		0.52 (0.74, 0.4)	0.34 (0.32, 0.35)	0.13 (0.2, 0.08)
**Random**			-0.16 (-0.38, -0.03)	-0.38 (-0.52, 0.3)
**Opposite**				-0.21 (-0.12, -0.26)
**Blue-bias**				

source types); and *(iii)* for differences within an experiment and between different source types (interaction).

For *(i)*, we pooled confidence ratings across different source types for each condition and performed a permutation test to check whether the two conditions differed in their mean confidence rating. We used permutation tests because the distributions of confidence ratings were not Gaussian. The sample sizes per group were $N_1 = 28224$ confidence reports for the full feedback condition, and $N_2 = 14784$ confidence reports for the noisy feedback condition. While the difference in means was statistically significant ($|\mu_1 - \mu_2| = 2.51$ with $p \ll 0.05$) the effect size was small (Cohen's d 0.17).

For *(ii)*, we pooled confidence ratings across the two different main conditions. We again used permutation tests to check for significant differences, but now between different source types. We, again, found statistically significant differences between all of the four source types. The pooled sample sizes were $N = 10752$ for each condition. We report the full values in Table A. The strongest effect was between the helpful and the random source. The weakest was between helpful and blue-bias. This exactly mirrors the general findings of the main study.

The analysis of interaction effects *(iii)* reveals that the largest effect (in both experiments) occurred between the helpful and random source. Notably, the findings in the noisy condition without feedback were particularly interesting. In contrast to the full feedback condition, there was nearly no effect between the helpful and blue-bias source, as well as between the opposite and random source. However, the differences of helpful and blue-bias showed a moderate effect when compared to the opposite and random conditions (magnitude of Cohens' d > 0.25, c.f. Table A).

Noisy feedback condition: responses to independent council

In the noisy feedback condition, participants should use the independent council as a trustworthy, albeit noisy, feedback mechanism. This means that they should respond positively to its information, increasing their confidence in blue when they see more blue endorsements. This shift should be visible regardless of whether participants played with the helpful, the random, opposite or blue-biased source. This was indeed the case, as we show in Fig. B. There, we plot the average changes of mind, that is the difference between the final confidence after seeing the independent council and the initial confidence after only seeing the news station. Participants shifted their beliefs increasingly towards the blue option the more blue endorsements they saw in the independent council. We note that there are differences in the strength of this shift depending on the news source that participants played with – but that this is due to the joint distributions between the respective news source and independent council, and the accompanying ceiling effects: For example, in the case of the helpful source, participants would have likely already seen a large number of blue endorsements, leaving little room to shift more towards blue when the independent council also favored blue.

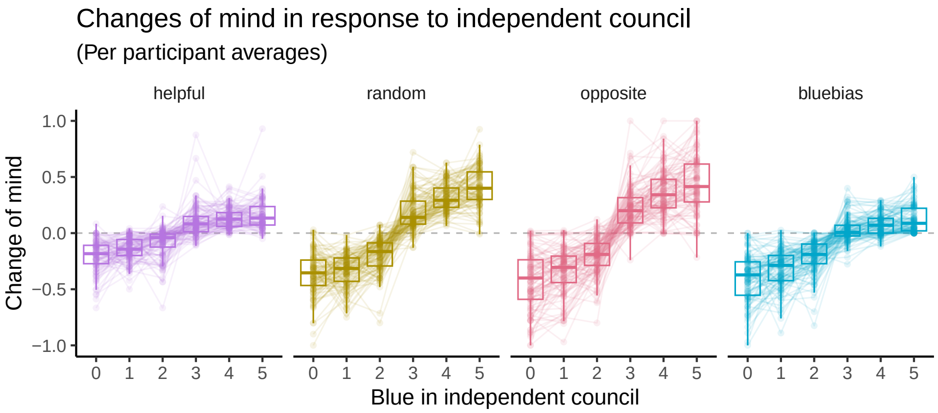

Fig B. Participants used the independent council as a sensible feedback signal and changed their mind to it accordingly in the noisy feedback version. We plot the changes of mind in response to the independent council as a function of the number of blue endorsement in the independent council. We computed the changes of mind by subtracting the initial confidence (after seeing only the news station) from the final confidence (after additionally seeing the news station). We plot individual averages (lines and dots) and distributions over these (boxplots).

Questionnaires and factor analysis

After finishing the main task, participants answered a number of questionnaires related to real-world trust and media consumption patterns:

- The trust question from the World Value Survey. Specifically, participants answered "Generally speaking, would you say that most people can be trusted or that you need to be very careful in dealing with people?" on a scale from 0 to 100.
- The epistemic trust questionnaire from Campbell et al. (2). This questionnaire contains three subscales: *Epistemic trust*: A penchant to be open to social learning in "benign social situations", and to do so adaptively. *Epistemic mistrust:* Tendency to view any information provided by others as unreliable and malign and the resulting hesistancy to rely on such information. *Epistemic credulity:* Absence of "vigilance" towards others, and the resulting tendency to fall prey to deception and misinformation.
- Questions assessing news consumption frequency. Participants were asked "How often did you use the following types of sources to get news in the past week?" with regards to ten different news types, and could respond using four different options ("Never", "1 - 2 days", "3 - 4 days", "5 - 7 days").[1]
- A questionnaire about the specific news sources participants consumed via a simple yes and no response ("Which of the following media outlets (if any) do you follow to get news?"). [2]

[1]The news types were: "Cable television news (e.g. CNN, Fox News, MSNBC)", "National network TV news (e.g. ABC, CBS, NBC)", "Local television news", "Social media (e.g. Facebook, Twitter, TikTok, Reddit)", "Podcasts", "Blogs", "Public radio (e.g. NPR)", "Talk radio", "News websites or apps", "Print newspapers"

[2]We assessed the following news organizations: ABC News, Breitbart, CBS News, CNN, Fox News, MSNBC, NBC News, New York Times, NPR, Wall Street Journal, Washington Post, USA Today, Other; and the following social media sources: Facebook, Instagram, Twitter, WhatsApp, Snapchat, TikTok, YouTube, Reddit, LinkedIn, Other

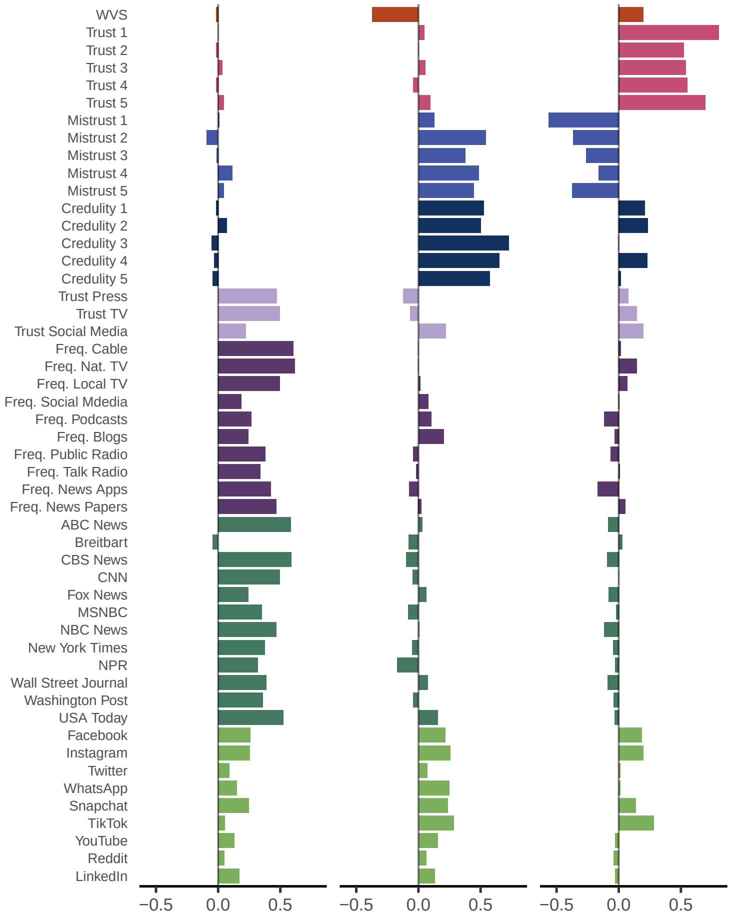

Fig C. Factor analysis of questionnaire data. Factor loadings for the three factors.

- A questionnaire assessing participants' trust in different types of media ("To what extent do you trust the information that comes from the following?": "The press (e.g. news apps, newspapers)", "Television", "Social media"). Participants could answer using on a five-point scale ("None at all", "A little", "A moderate amount", "A lot", "A great deal").

We conducted an exploratory factor analysis on the total set of 50 questions. The number of factors was determined using the Cattell-Nelson-Gorsuch test. We used maximum-likelihood estimation with a an oblique rotation (oblimin). We discuss the results of the factor analysis in the main text and the factor loadings are plotted in Fig C.

S3 Modelling

Optimal model: Full feedback learning

In the full feedback version of the task, we can model an optimal agent's posterior over the sources' probabilities as two independent beta distributions that both are governed by an α and β parameter. We here drop the subscript I indicating the source but note that this learning process proceeds individually and independently for all sources:

$$b_t|\mathcal{H}_t \sim Beta(\alpha_{b,t}, \beta_{b,t}) \tag{1}$$
$$g_t|\mathcal{H}_t \sim Beta(\alpha_{g,t}, \beta_{g,t}) \tag{2}$$

To update its estimate of the two probabilities b_t and g_t, the agent computes the following posterior (here only for b_t but g_t proceeds analogously):

$$p(b_{t+1}|X_t, s_t, \mathcal{H}_t) \propto P(X_t|s; b_t)p(b_t|\mathcal{H}_t)\mathbb{1}(s_t = blue) \tag{3}$$

Thereby, $P(X_t|s; b_t)$ is again a binomial likelihood, and $p(b_t|\mathcal{H}_t)$ the prior distribution over b_t. The indicator $\mathbb{1}(s_t = blue) \in \{0, 1\}$ means that we only update our beliefs about b_t when we are in a blue state. Because we are updating a beta-distribution with a binomial likelihood, this update takes the form of a regular beta-update, so that when $s_t = blue$ (and vice-versa for green):

$$\alpha_{b,t+1} = \alpha_{b,t} + X_t \tag{4}$$
$$\beta_{b,t+1} = \beta_{b,t} + n - X_t \tag{5}$$

The four parameters $\alpha_{b,t}$, $\beta_{b,t}$ and $\alpha_{g,t}$, $\beta_{g,t}$ are thus sufficient statistics for the qualities of the news sources and are the only quantities that need to be carried forward between trials.

Optimal model: Noisy feedback learning

We here outline the computations underlying the agents' update over the sources parameters b_I and g_I in more detail. As we discussed, this is done via an update on the joint distribution of these two parameters. We again drop the source subscript I for convenience:

$$p(b_{t+1}, g_{t+1}|Y_t, X_t,) = \sum_{s_t} p(b_{t+1}, g_{t+1}, s_t|Y_t, X_t,) \tag{6}$$
$$\propto \sum_{s_t} p(Y_t, X_t|s_t; b_t, g_t)p(b_t, g_t, s_t|) \tag{7}$$
$$\propto \sum_{s_t} P(X_t|s_t; b_t, g_t)p(b_t, g_t|)P(Y_t|s_t) \tag{8}$$
$$\propto [B(X_t; n, b_t)B(Y_t; n, b_Y)+ \tag{9}$$
$$B(X_t; n; 1 - g_t)B(Y_t; n, 1 - b_Y)]\, p(b_t, g_t|) \tag{10}$$

Essentially, the agent updates its prior belief $p(b_t, g_t|)$ with the likelihoods of X_t, given this prior belief, but weighted by the likelihood obtained from the noisy feedback sample Y_t, all while marginalizing out the state.

Optimal model: Inferring the ground-truth

After seeing the news station, in both the full feedback and the noisy feedback case, the model updates its beliefs equivalently, marginalizing over its current beliefs about the sources, $p(b_{t,I}, g_{t,I}|)$. Specifically, it computes the posterior as follows, here for $s_t = blue$ assuming a flat prior over states $p(s_t) = 0.5$ and dropping the subscript I for convenience.

$$P(s_t = blue|X_t,) = \frac{P(X_t|s_t = blue,)}{P(X_t|s_t = blue,) + P(X_t|s_t = green,)} \tag{11}$$

$$= \int_{b_t}\int_{g_t} p(b_t, g_t) \frac{P(X_t|s_t = blue; b_t, g_t)}{P(X_t|s_t = blue; b_t, g_t) + P(X_t|s_t = green; b_t, g_t)} db_t dg_t \tag{12}$$

$$= \frac{\int_{b_t} P(X_t|s_t = blue; b_t) p(b_t) db_t}{\int_{b_t} P(X_t|s_t = blue; b_t) p(b_t) db_t + \int_{g_t} P(X_t|s_t = green; g_t) p(g_t) dg_t} \tag{13}$$

Thereby, $P(X_t|s_t = \text{blue}; b_t)$ is simply the binomial likelihood $B(X_t; n, b_t)$ and in the noisy feedback version $p(b_t)$ is just the marginal distribution $p(b_t) = \int_{g_t} p(b_t, g_t)$ (and vice-versa for $s_t = green$ and g_t).

In the noisy feedback version, this initial posterior is then integrated with the information from the independent council Y_t to form a final posterior (with no closed form update):

$$P(s_t|X_t, Y_t; b_Y,) \propto P(Y_t|s_t; b_Y) P(s_t|X_t,) \tag{14}$$

Model fitting: Parameters

To capture participant idiosyncrasies in learning and confidence reporting, we implemented an eight parameter version of the optimal model, and fit these parameters to participants' behavior using a ninth noise parameter. These parameters are partially shared between the two experiments and models, and partially individual.

We begin by outlining the **shared parameters**. A first shared parameter by the models relates to the way participants report their confidence in a potentially non-linear way. Specifically, we let the confidence be scaled non-linearly using the parameter κ. Here, we denote the reported confidence as $\hat{c_t}$ and the original confidence $c_t \in \{P(s_t = 0|X_t;), P(s_t = 0|X_t, Y_t; b_Y, b_t, g_t)\}$:

$$\hat{c_t} = c_t^{\kappa} / (c_t^{\kappa} + (1 - c_t)^{\kappa}) \tag{15}$$

Crucially, this transformation only impacts the confidence report but not the probabilistic estimates that get made and carried forward by the model.

Another set of four parameters relate to the priors about the source probabilities, $p(b_{t=0}, g_{t=0})$, an agent might have when approaching this task. We implemented this by instantiating the initial beliefs of the agent as beta distributions with the parameters $\alpha_{b,0}, \beta_{b,0}, \alpha_{g,0}, \beta_{g,0}$, so that agents had the following prior

$$b_{t=0} \sim Beta(\alpha_{b,0}, \beta_{b,0}) \tag{16}$$

$$g_{t=0} \sim Beta(\alpha_{g,0}, \beta_{g,0}) \tag{17}$$

In the **full feedback experiment**, we consider three more parameters: Specifically, agents might have a tendency towards updating their confidence in a more or less optimistic manner. We capture this by weighting the beta-binomial update by two parameters ω_α and ω_β, so that each updates proceeds as following, here for when $s_t = blue$:

$$\alpha_{b,t+1} = \alpha_{b,t} + \omega_\alpha X_I \tag{18}$$

$$\beta_{b,t+1} = \beta_{b,t} + \omega_\beta (n - X_I) \tag{19}$$

Table B. Summary of fitted model parameters for the Bayesian Model.

$\alpha_{b,0}, \beta_{b,0}, \alpha_{g,0}, \beta_{g,0}$	parameters of Beta-priors (shared)
κ	parameter of non-linear confidence scaling (shared)
$\omega_\alpha, \omega_\beta$	weighting of beta-binomial update (full feedback)
λ	forgetting rate (full feedback)
ν	mixing factor of biased belief update and forgetting (noisy)
$\alpha_{forget}, \beta_{forget}$	forgetting distribution (noisy)

Table C. Fitted parameter statistics for the Bayesian model.

Parameter	Mean (ffb)	Std (ffb)	Mean (Noisy)	Std (Noisy)
beta priors blue	0.56	0.13	0.66	0.18
beta priors green	0.63	0.12	0.70	0.14
κ	3.83	3.28	1.58	2.55
ω_α	1.08	0.47	n.a.	n.a.
ω_β	1.06	0.42	n.a.	n.a.
λ	0.04	0.11	n.a.	n.a.
ν	n.a.	n.a.	0.25	0.30
α_{forget}	n.a.	n.a.	28.33	13.44
β_{forget}	n.a.	n.a.	19.82	11.65

Furthermore, agents might have a tendency to forget (towards the uniform $Beta(1,1)$), which we implement via a forgetting rate parameter λ. That is, between each trial noise gets added to the parameter estimates and there is a regression to the mean (for both *green* and *blue* parameters, hence dropping the colour subscript here):

$$\alpha'_t = (1-\lambda)\alpha_t + \lambda \tag{20}$$
$$\beta'_t = (1-\lambda)\beta_t + \lambda \tag{21}$$

In the **noisy feedback model**, we consider a similar set of phenomena. However, this is implemented somewhat differently because of the joint belief distribution. Specifically, we also implement a forgetting parameter but instead of merely adding noise and forgetting towards a uniform distribution, we forget towards a specific distribution, thereby implementing both a biased belief update and forgetting. We call this forgetting parameter ν. Specifically, ν weights the current two-dimensional belief state $p(b_t, g_t)$ with a second two-dimensional distribution $p_{\text{forget}}(b, g)$ between trials:

$$p(b_t, g_t)'_t = (1-\nu) * p(b_t, g_t) + \nu * p_{\text{forget}}(b, g) \tag{22}$$

We parameterize $p_{\text{forget}}(b, g)$ to be a 2-dimensional distribution made out of the outer product of two beta distributions both sharing the two parameters α_{forget} and β_{forget}

$$p_{\text{forget}}(b, g) = Beta(\alpha_{\text{forget}}, \beta_{\text{forget}})Beta(\alpha_{\text{forget}}, \beta_{\text{forget}}). \tag{23}$$

Table B provides an overview about all fitted parameters for quick reference. Further, Table C shows statistics of the fitted parameters.

Model fitting: Parameter estimation

We fit the model to participants' data using maximum likelihood estimation via differential evolution implemented in the DEOptim package in R. Participants were fit individually with one set of parameters for all sources. To obtain a likelihood for our

data given a parameterized model, we fit the confidence ratings in the data $c_{t,D}$ as being sampled from a Gaussian distribution centered around the model predictions given parameters θ, $c_{t,M|\theta}$ and a standard deviation σ_c:

$$c_{t,D} \sim \mathcal{N}(c_{t,M|\theta}, \sigma_c) \tag{24}$$

The noise term σ_c was a free parameter in our fitting, resulting in a total of 9 parameters.

In both experiments, we fit the initial confidence reported after participants had seen the news station. In the noisy feedback experiment, we additionally fit the second confidences participant reported after having seen the independent council. In both versions, we also fit the 6 probe trials. The probe trials were fit using the frozen belief state after the final main block trial, without any additional forgetting.

S4 Analysis details

Regression analysis

To obtain slopes and intercepts from our data and model, we fit logistic regressions. To deal with the limited data per regression and participants whose responses showed perfect separation, we used Bayesian generalized linear models implemented in the "arm" package in R using the default Cauchy priors. We fit the following regression analyses:

- For the probe trial analysis, we fit four individual regressions per participant, each time fitting their individual six responses per source
- For the pooled analysis split by block quarter, we pooled all participants' data, and then split it into bins of 7 trials, fitting one regression per source and block quarter
- For the analysis investigating participants' individual learning progress, we split their data into the first and last 14 trials, and fit one regression per half and source.

We ran these equivalent regressions on the predictions made by the model fit to participant data. To do so, we simulated behavior from the model using the fit parameters. We used the mean confidence prediction of the fit model with no additional noise. In all regressions, we subtracted 2.5 from the number of of blue endorsements, so that an unbiased response would have a intercept of 0.

Statistical analysis of main task

To further analyze participant behavior, we conducted the following statistical analyses, both for the full and noisy feedback version:

- To investigate whether the slopes and intercepts differed from each other in the probe trials between news sources, we conducted an ANOVA predicting the slope (or intercept, respectively) from the source type. We conducted Tukey's honest significance test to compare the individual means.
- To investigated whether participant's slopes and intercept differed from 0, we conducted one-sample t-tests.
- To investigate quality of fit between probe trial slopes and intercepts computed for the data and the model predictions, we computed Pearson's R.

- To investigate effects of trial and news source on participants' accuracy, we set up a logistic regression predicting, on a trial-by-trial basis, participants' accuracy from the trial number (within a block) and the source, as well as their interaction. To test for the significant effects of these predictors, we conducted χ^2 tests.
- To investigate whether slopes and intercepts differed between block halves and news sources, we conducted an ANOVA predicting the slope/intercept from the source type and the block half. We conducted Tukey's honest significance test to compare the individual means.

We ran the following analyses to compare the two conditions and the optimal model:

- To compare the accuracy achieved between the optimal model and the data, we set up a logistic regression predicting, on a trial-by-trial basis, participants' accuracy from the trial number (within a block), as well as from the data type (optimal model or data) and the interaction of these two predictors. To test for the significant effect of these predictors, we conducted a χ^2 tests.
- To compare the slopes achieved by the models, we set up an ANOVA predicting the probe trial slopes from the experiment condition and the news source. We conducted Tukey's honest significance test to compare the individual means.

Trust and improvement ratings

To investigate the different types of responses to the post-block trust and improvement questionnaires, we set up an ANOVA predicting the response from the source type and the response type (trust/improve). We conducted Tukey's honest significance test to compare the individual means.

To check how participants' trust and improvement ratings were related to their task behavior, we set up regressions predicting the respective rating from the slopes inferred as above from the participants' second half responses. We set up three regressions per relationship, (1) one predicting the rating linearly from the slopes, (2) another predicting the ratings from a quadratic function of the slopes, and (3) a final using both a linear and quadratic function. We compared the quality of these fits using the regressions' respective Bayesian Information Criteria, reported in Table D. Slopes and intercepts of the winning models all reached statistical significance ($p < 0.05$).

S5 Additional analyses

Individual belief states

Figures 4 and 8 showed the modeled belief states of individuals as an average of b_t and g_t. To provide more detail, we show the trajectories of the separate beliefs in Figure D.

Mixed effects Bayesian logistic regression

For further validation, we fit mixed effects Bayesian logistic regression. We constructed the model with group level slope and intercept parameters, as well as participant level slopes for the logistic regressors. For fitting, we used the glmmTMB package[3] in R. Including a participant level intercept was not significant. While the mixed effects logistic regression provided a better BIC in general, yet we used the quarterly analysis in the main text to visualize the learning progress over time.

[3]https://cran.r-project.org/web/packages/glmmTMB

Table D. Comparison of model fit for the post-block trust and improvement ratings: BICs for regression models predicting the post-block trust questionnaires from the betas fit to participants' responses in the second half of a block.

DV	News Sources	Model	BIC
Trust	All but opposite	Linear	**1585.6531**
Trust	All but opposite	Quadratic	1589.4885
Trust	All but opposite	Linear and quadratic	1590.7492
Trust	Opposite	Linear	**690.0235**
Trust	Opposite	Quadratic	696.8924
Trust	Opposite	Linear and quadratic	694.5620
Improve	All but opposite	Linear	**1602.8504**
Improve	All but opposite	Quadratic	1610.0373
Improve	All but opposite	Linear and quadratic	1608.9850
Improve	Opposite	Linear	720.3958
Improve	Opposite	Quadratic	718.0166
Improve	Opposite	Linear and quadratic	**717.8379**

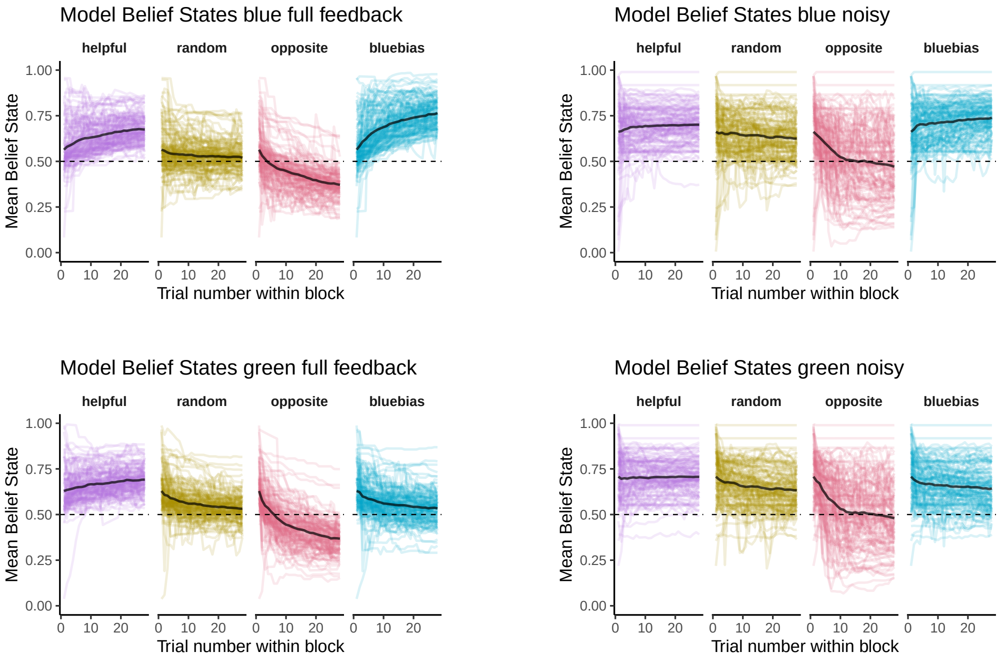

(a) full feedback condition **(b)** noisy condition

Fig D. Belief states reported separately for blue and green.

Table E. Group-level parameters of mixed effects logistic regression on the participants learning trajectories. The stars indicate statistical significance against 5%.

Condition	Source Type	Slope	Intercept
full feedback	helpful	1.95*	-0.02
full feedback	random	0.54*	-0.01
full feedback	opposite	-0.66*	-0.01
full feedback	blue-bias	1.31*	-0.23*
noisy	helpful	1.28*	0.05
noisy	random	1.04*	0.01
noisy	opposite	0.25*	-0.03
noisy	blue-bias	1.17*	-0.05

Most importantly, the mixed effects model agreed with our main findings using the quarter-based and half-based analyses of learning. That is, the group level slopes were significant for both conditions for all four source types and had the same signs as in the quarter-based analysis. The blue-bias source had a significant intercept in the full feedback condition, but not in the noisy condition. We report all parameter values in Table E.

Comparison to Rescorla Wagner learning

In addition, we compare the Bayesian model against a structurally simpler model - a Rescorla-Wagner (RW) rule, since this operates according to related, but less sophisticated, incremental learning principles. The main difference between RW and Bayesian learning is the fixed learning rate in RW. Learning in the Bayesian model is fast initially (when epistemic uncertainty dominates) and slow ultimately (when aleatoric uncertainty dominates). Thus, if the participants are more Bayesian in their adaptation, then we can expect the RW to fit poorly, but in a way that might be hard to predict.

For the RW, to allow for suitable generalization, we model Q values of observation x using a quadratic form as $Q(x) = w_0 + w_1 x + w_2 x^2$ and apply the logistic function to transform those Q values to probabilities. We found that a cubic term afforded no extra predictive power, but it was not practical to test the many other possible functional forms. Nevertheless, to provide RW with as much flexibility as possible, we allowed separate learning rates $\lambda_0, \lambda_1, \lambda_2$ for each parameter, per participant. Figure G shows fitted learning rates for those RW fits.

To compare the learning behavior of the RW model together with the Bayesian model and our participants, we conducted an analysis which parallels that in the main paper: We simulated learning trajectories of the computational models, having fit any parameters to the behavior of the subjects. We then split the simulated trajectories and the behavior of the participants into two halves (akin to our procedure in the main part of the paper) and fit psychometric slopes and intercepts using Bayesian logistic regression as before. For each source type in each condition we used an ANOVA test to assess whether the slopes and intercepts were significantly different between the models and the data[4]. We found that the RW model had significantly lower slopes for nearly all source types and block halves in the full feedback condition (Figure E). This suggests that the RW model lags behind the participants' (and the Bayesian model's) learning speed, likely due to its fixed learning rate. For the intercept, we only found a significant difference in the second block half in the blue-bias condition. The RW model's lower intercept compared to the participants (and the Bayesian model) suggests an

[4]We then used pairwise t-tests to examine the difference was in the RW model.

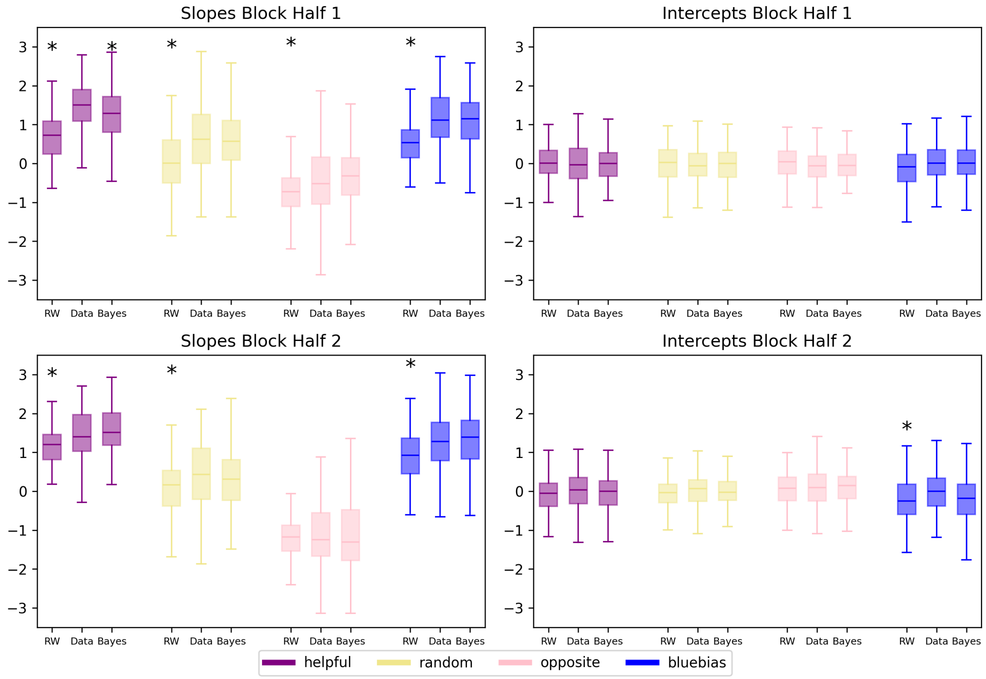

Fig E. Model comparison in the full feedback condition. The asterisk indicates a significant ANOVA test for the source type, and marks whether the respective model deviates from the participant data according to a t-test.

overestimation.

We report the findings for the noisy condition in Figure F. The results for the fitted intercepts agree with the full feedback version. The results for the slopes are more challenging to interpret, likely due to the additional noise. The slopes were lower for the RW than the participants in many conditions, albeit higher for the helpful and blue-bias source in the second half. Nevertheless, even when the ANOVA test implied significant differences across the three learning curves, the Bayesian model only significantly deviated from that of the participants in the first block of the helpful source.

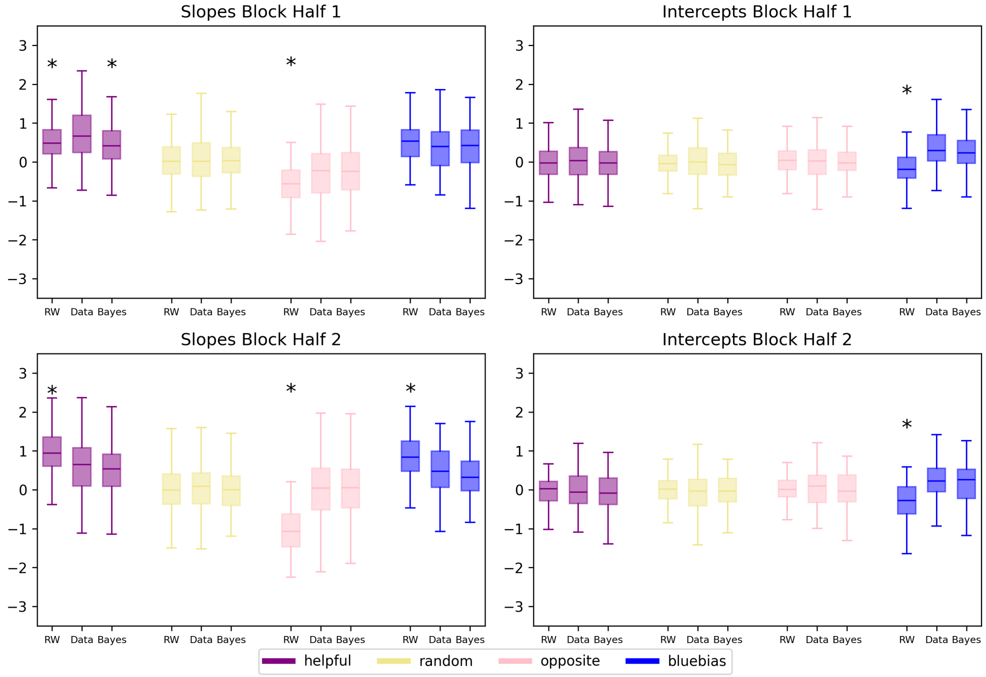

Fig F. Model comparison in the noisy condition. The asterisk indicates a significant ANOVA test for the source type, and marks whether the respective model deviates from the participant data according to a *t*-test.

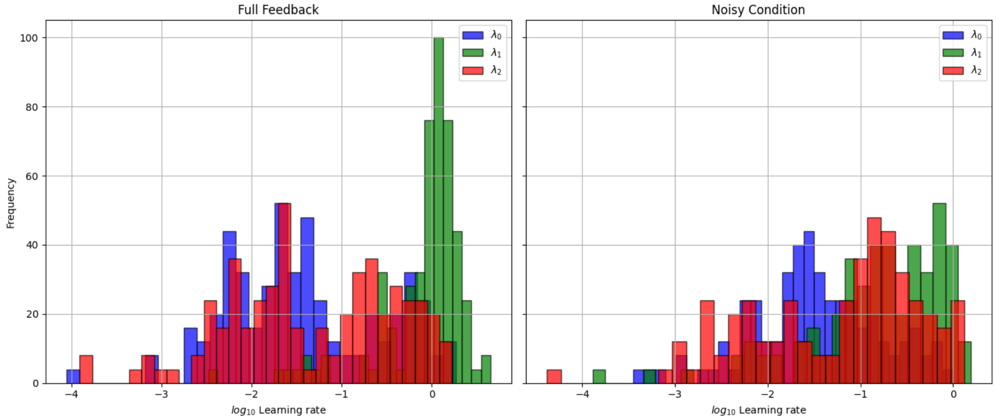

Fig G. Fitted learning rates of the Rescorla Wagner model

List of Figure Legends

Figure	Description
**Fig. A**	**Demographics.** (A-C) Experiment 1. (D-F) Experiment 2.
**Fig. B**	**Participants used the independent council as a sensible feedback signal and changed their mind to it accordingly in the noisy feedback version.** We plot the changes of mind in response to the independent council as a function of the number of blue endorsement in the independent council. We computed the changes of mind by subtracting the initial confidence (after seeing only the news station) from the final confidence (after additionally seeing the news station). We plot individual averages (lines and dots) and distributions over these (boxplots).
**Fig. C**	**Factor analysis of questionnaire data.** Factor loadings for the three factors.
**Fig. D**	Belief states reported separately for blue and green.
**Fig. E**	Model comparison in the full feedback condition. The asterisk indicates a significant ANOVA test for the source type, and marks whether the respective model deviates from the participant data according to a t-test.
**Fig. F**	Model comparison in the noisy condition. The asterisk indicates a significant ANOVA test for the source type, and marks whether the respective model deviates from the participant data according to a t-test.
**Fig. G**	Fitted learning rates of the Rescorla-Wagner model.

List of Table Legends

Table	Description
**Table A**	Cohen's d values between different source types. We report the value pooled across full feedback and noisy, as well as the individual values (full feedback, noisy).
**Table B**	Summary of fitted model parameters for the Bayesian model.
**Table C**	Statistics for fitted parameters of the Bayesian model.
**Table D**	Comparison of model fit for post-block trust and improvement ratings: Bayesian Information Criteria (BICs) for regression models predicting post-block trust ratings based on betas fitted to participants' responses in the second half of a block.
**Table E**	Group-level parameters of a mixed-effects logistic regression on participants' learning trajectories.

References

1. Staël von Holstein CAS. Measurement of subjective probability. Acta Psychologica. 1970;34:146–159. doi:10.1016/0001-6918(70)90013-2.

2. Campbell C, Tanzer M, Saunders R, Booker T, Allison E, Li E, et al. Development

and validation of a self-report measure of epistemic trust. PLoS ONE. 2021;16(4 April):1–21. doi:10.1371/journal.pone.0250264.
