# Supplementary material for: Mechanisms of mistrust: A Bayesian account of misinformation learning
Source: PLoS Comput Biol. 2025 May 14;21(5):e1012814. doi: 10.1371/journal.pcbi.1012814 (PMC12077715; doi:10.1371/journal.pcbi.1012814)
Supplement: Attachment — Submitted filename: 01-misinformation_Response_Letter.pdf [file pcbi.1012814.s002.pdf]

Dear Profs. Mathys and Serre,

Thank you very much for sending us the reviews of our paper 'Mechanisms of Mistrust: A Bayesian Account of Misinformation Learning'. We are very grateful to the reviewers for their advice and opinions. We have attempted to address all the points, and detail our response below (reviewer comments in blue; our response in black; changes to the paper in red). We also attach an updated manuscript with main changes hightlighted in red.

We hope that you will agree that the paper has been substantially strengthened with these changes, and look forward to your further thoughts.

You will notice that we have added an author – Yannick Streicher – who has contributed substantially to the writing of this revision, and its underlying analyses.

Yours sincerely,

Lion Schulz, Yannick Streicher, Eric Schulz, Rahul Bhui, and Peter Dayan.

Responses to Reviewer 1

Evaluation

> **Reviewer:**
> *Reviewer #1: Schulz et al. addressed the mechanisms of trust/mistrust using a Bayesian learning framework and carefully designed experiments. Across two studies, they examined how participants use information from (a) helpful, (b) random, (c) opposite, and (d) biased sources, after receiving full (Study 1) and partial (Study 2) feedback. The analysis followed the psychometrics function as motivated by the literature. The results mostly followed the theoretical predictions, and there are interesting differences between full vs partial feedback. This manuscript is very clear and well-written, and the topic is important and timely, especially nowadays information/misinformation on social media plays a key role in everyday life. I only have a handful of clarification questions that hopefully will improve the paper.*

We thank the reviewer for this positive evaluation of our paper and are grateful for their suggestions that we now address in full.

Point 1

> **Reviewer:** *What is the meaning of the three small triangles in Fig1B? Does it indicate a news station? It'd be great to make it explicit (also for Fig 5).*

We apologise for not making this clear. They do indeed represent the particular news station. The text now says (Line 144):

> In the task, different news stations were represented via different abstract symbols (here, the pattern of white dots in the purple square in Figure 1B). Each news station had an individual pattern to individuate it, making clear to participants when they were interacting with a novel news station. However, the identity of each news station stayed hidden.

Point 2

> **Reviewer:** *In line 160, it would be helpful to define the meaning of Y already here, rather than later (line 168).*

Thanks for the suggestion. Line 142 now says:

> Participants saw a piece of information $X_{I,t}$ from an imaginary news station I pertaining to the quality of the policies, along with a complete or partial feedback signal, Y_t

and Line 214 now says:

> As we mentioned in the introduction, at least some feedback signal or additional knowledge is necessary for this learning to succeed [...]. Here, we denote this feedback as Y.

Point 3

> **Reviewer:** *line 175 "the agent combines its prior [...] to form an updated belief about the two parameters [...]" It seems that the inferred two parameters are b and g; but following the reasoning of this paragraph, the two parameters might refer to the slope and intercept in the psychometric function? Please clarify.*

Sorry for the confusion. We reworded this as (Line 222):

> Specifically, the agent combines its prior estimate about the parameters of the sources, $p(b_t, g_t \mid \mathcal{H}_t)$, and current information X_t and Y_t to form an updated belief about the two parameters (again dropping I).

Point 4

> **Reviewer:** *In general, what are the parameters in the model? Indeed more details are provided in the SI, but it would be very helpful for readers to clearly see the notions and meanings of all free parameters, eg, by including a table.*

Thanks for the suggestion. We included the following table in the supplement, to make the parameterization transparent, and to have a quick reference. Line 876 now says:

Table 2 provides an overview about all fitted parameters for quick reference.

Table 2: Summary of Fitted Model Parameters for the Bayesian Model.

$\alpha_{b,0}, \beta_{b,0}, \alpha_{g,0}, \beta_{g,0}$	parameters of Beta-priors (shared)
κ	parameter of non-linear confidence scaling (shared)
$\omega_\alpha, \omega_\beta$	weighting of beta-binomial update (full feedback)
λ	forgetting rate (full feedback)
ν	mixing factor of biased belief update and forgetting (noisy)
$\alpha_{forget}, \beta_{forget}$	forgetting distribution (noisy)

Point 5

> **Reviewer:** *I am not entirely convinced by the twice data split. Isn't it the case that blocks 1-2 are the first half of the experiment, and blocks 3-4 are the second half of the experiment? So technically, the data is analyzed twice, just by different split criteria? This seems arbitrary, and knowing the data from 4 blocks, one could already infer the results with the half-split. I would rather leave out the half-split results.*

This is an important point. The two analyses indeed both cover the entire experiment. However, the quarter-based analysis concerns the whole population, whereas the half-based analysis allows us to look at individual differences.

To amplify: since each participant only performed 28 trials per source, a quarter-based analysis at the individual level would be too noisy. Therefore, we pool participants for the quarter-analysis to draw conclusions about the average behavior across the population. For statements about the psychometrics of

individuals, we use the half-based analysis, which has sufficient trials per participant.

To clarify our approach, we added detail at the corresponding paragraphs in the text (Line 359):

> To investigate whether these learning patterns held on an individual level, we next fit logistic regressions individually to the data. ~~, although this time split by block half rather than quarter to ensure a sufficiently large sample per regression~~To ensure a sufficiently large sample size per regression, we split each participant's 28 trials into two halves.

Point 6

> **Reviewer:** *For the model belief dynamics (Fig 4E, 8E), it would be helpful to actually plot the beliefs for blue and green separately, rather than the average. This matters because the design is framed with respect to the blue information, particularly for the biased information source. Hence it'd be great to see the beliefs for green and blue separately.*

Thank you for this suggestion. Indeed, we agree that it is helpful to look at the beliefs in separation. We provide versions of plots 4E and 8E for blue and green separately in Figure 14 and add it to the Appendix of the paper. We would slightly beg to differ that the task is framed in terms of the blue information – the instructions and the response modalities are neutral (otherwise there could have been an untoward bias). It is just that the descriptions in the paper are framed in terms of blue, for convenience.

Line 954 now says:

> Figures 4 and 8 showed the modeled belief states of individuals as an average of b_t and g_t. To provide more detail, we show the trajectories of the separate beliefs in Figure 14.

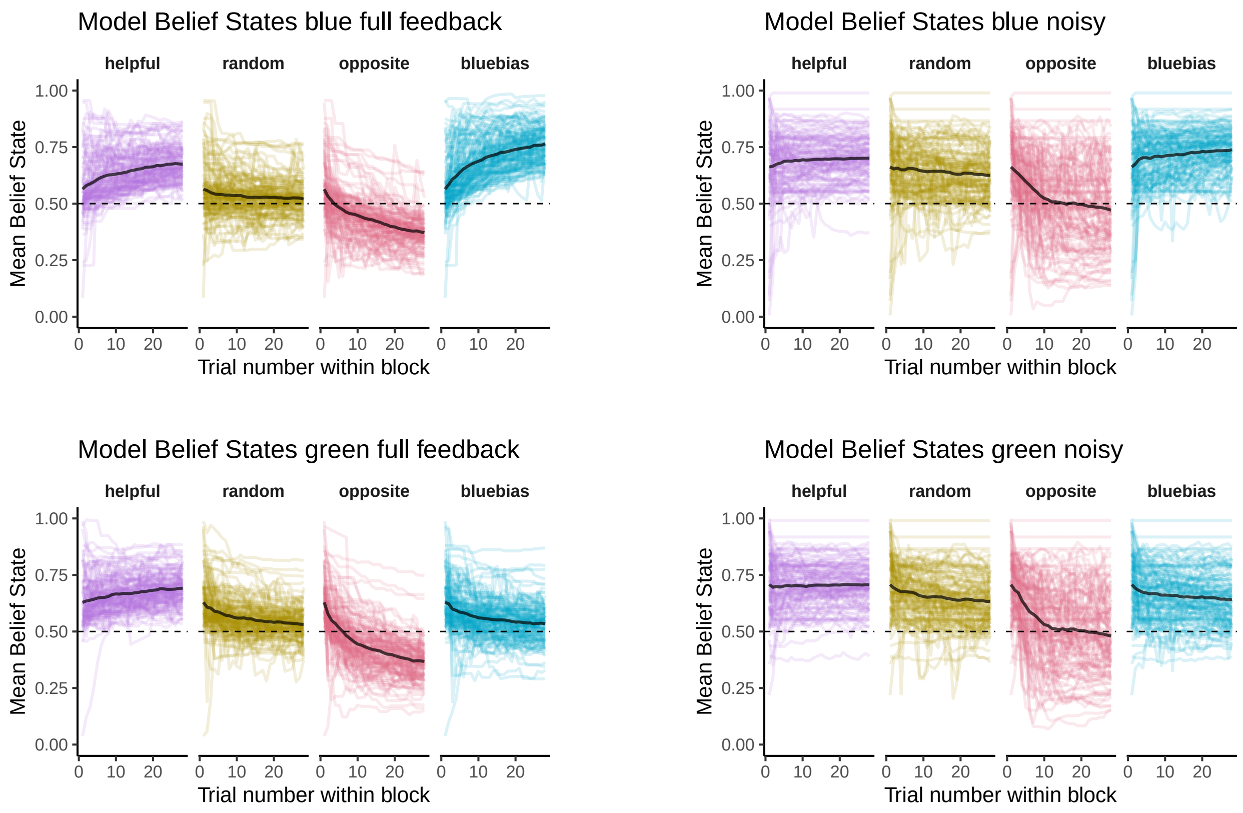

(a) full feedback condition

(b) noisy condition

Figure 14: Belief states reported separately for blue and green.

Point 7

Reviewer: *I was rather intrigued by the differences in the priors between the two studies. Indeed, in both cases, the prior is more positive (above 0.5), but clearly, a large proportion of participants' prior in Study 1 is around 0.5, whereas most participants' prior in Study 2 is about 0.75. It would be useful to provide some interpretation (around Line 561).*

Thank you for the suggestion. We suggest that the main reason for the difference is that in the noisy feedback condition, we explicitly and carefully instructed the participants about the degree of helpfulness of the sources; whereas in the full feedback condition, we did not. The relevant text reads: 'Here, we set $b_Y = 0.75$. Participants were extensively informed about this quantity. We conducted analyses to ensure that participants used the independent council's feedback…'

Point 8

Reviewer: *Fig 10; in addition to the relationship between Trust/Improve and psychometric parameters, I wonder if Trust/Improve is associated with some parameters from the Bayesian learning model?*

Thank you for the suggestion. We actually did not expect to find a statistically significant correlation be-

tween single parameters of the Bayesian model and Trust/Improve; and indeed did not. We predicted this null result since Trust and Improve are designed to capture the effects of learning – and so what amounts to the output of the Bayesian model – rather than the input parameters. If the prior had been so extreme that no learning was possible, then this might have been different. However, as evident in the figures, this was not the case.

Responses to Reviewer 2

> **Reviewer:** *In their work 'Mechanisms of Mistrust: A Bayesian Account of Misinformation Learning' Schulz et al. study the way humans learn about the accuracy of information sources. They used a novel experimental paradigm and a computational Bayesian learning model to uncover this phenomenon. In two experiments, they presented a panel of opinions as a source of information, which could be accurate, non-informative, biased, or wrong (or misleading). They found that participants learn the relation between the information source and the real state of the world, as reflected in their estimations. When the outcome was ambiguous, and participants had to infer both the state of the world and the source accuracy, participants found it harder to estimate the bias and information content of the information source. Their work shows that participants prior about source accuracy, coupled with uncertain outcome, greatly affect estimation of source accuracy. This work presents compelling computational framework and experimental design and provides important evidence concerning the way people tackle uncertainty in social learning. I personally found the results from the random condition to be very interesting, as people tend to follow them as if they contain signal and fail to distinguish between noisy and coherent sources (especially when outcome is ambiguous). I think that the approach and methods are appropriate, and the paper is written in a clear way. I have a number of technical comments below, and one theoretical comment.*

We thank the reviewer for this positive evaluation and are grateful for their suggestions. Below, we address them in full.

Point 1

> **Reviewer:** *Theoretical comment: The authors frame their study as a way to evaluate trustworthiness of information sources, such as media outlets. However, unlike studies that directly use headlines from newspapers, social media or tv, or prepare stimuli that is very similar to these, they use a panel of opinions to convey an opinion. I am not concerned about this reduction, but I do think that this display brings some biases that the authors do not discuss. There is literature about majority opinion and its epistemic value and tendency to conform. The conformity works of Asch look at the effects of panel opinion, and many followers examined how different majorities affect individual decisions to comply with the group. This is even in cases where the group is known to be wrong, showing some motivation or bias to conform to the majority that may also be relevant to the current experimental design. Other works examine the epistemic value of majority, going back to Condorcet's jury theorem, and more recent works on informational cascades, economics research (Shmuel Nitzan's works for example), and in moral judgment (Lindström et al. (2018). common is moral). I think that this literature may be useful in framing and interpreting the results, and also to highlight the relevance of the experimental paradigm. Note that this may have some implications for your model, as these approaches assume that each panel member is an independent observation of the noisy world.*

Thank you for these very helpful pointers and references. We now include a discussion of these points:

Line 662:

> The role that sources play in helping people identify misinformation has been disputed. Research into immunizing people against misinformation indeed tries to highlight the nature and properties of the source (Breakstone et al., 2021; Hertwig & Engel, 2021). It has been reported that people generally tend to be able to assay the trustworthiness of news providers, at least in an US context (Pennycook & Rand, 2019). Furthermore, displaying explicit trustworthiness rating may lower people's propensity to share fake news (Celadin et al., 2023). However, other research (Dias et al., 2020) suggests that people can pay little attention to the sources of misinformation, making them more vulnerable to being misled. One prominent mechanism for fostering this vulnerability is the tendency of individuals to conform with majority opinion (Asch, 1951). Even in the simplified setting of our experiment, participants may experience the need to align their beliefs with the majority opinion (represented, e.g., by the council of experts), potentially putting lower weight on their own assessment of the trustworthiness of sources. This bias is reflected in the priors observed in the experiment, which suggest an inherent assumption that the majority is right. This bias is often (Austen-Smith & Banks, 1996; Lindström et al., 2018; Nitzan, 2009), though not always, useful – a fact that malign actors can exploit.

Point 2

> **Reviewer:** *[...] In addition, the current discussion linking the learning model to theory of mind seems a little forced. For example, it is not clear what one makes of the opposite group – it is not likely to face a source that faithfully tells you the opposite of the real state. What kind of theory of mind is at work here? In the sense that inferring the underlying distribution is a model of the source I agree that this is indeed the case, but I find that this link on its own is not very fruitful for understanding the way people infer about sources of information in its current state.*

Thank you for this comment. We apologise if the link to theory of mind (ToM) appeared to be overemphasised in the original version – we now outline why we think it important.

The key question for a consumer of information who is concerned about misinformation is motive – how a source could benefit from fooling the consumer. This question is exactly the one that is addressed in theories of mind such as the interactive POMDP that we cite. It also underlies a rigorous approach to 'cheap-talk' – as noted early in the paper.

It is true that the 'opposite' source is a little contrived in our circumstance; but we can see that it is still experimentally fecund - as in the difference between the learning in the case of full and partial information.

We edited the relevant passage (Line 676) to read:

> The inferences our participants and models draw about news providers can be understood as a basic form of theory of mind [...]: In essence, ~~they~~ participants reason and learn about how the sources report evidence associated with the ground truth by inverting their model of the sources using Bayesian inference. This type of inverse reinforcement learning has been posited as a key computational substrate for shallow forms of Theory of Mind (ToM) (Jara-Ettinger et al., 2016). More complex forms of theory of mind can also take into account the beliefs and intentions of other agents, and those other agents' recursive reasoning about the participants themselves (Gmytrasiewicz & Doshi, 2005). ~~The way we deal~~ In broader terms, the ability to deal with ~~those~~ such intentions ~~is particularly important, both~~ plays a signficant role in the consumption and interpretation of media and in psychopathology. When we suspect a source might profit from misleading us, we become more skeptical towards it, and its messages might even backfire (Bhui & Gershman, 2020) or be ignored as cheap talk (Farrell & Matthew, 1996). Computationally, this can be modelled using recursive theory of mind, where a sender tries to hack the inference process of a receiver and where this receiver might try to infer about such a hacking scheme and defend against it (Alon, Schulz, Rosenschein, & Dayan, 2023; Gmytrasiewicz & Doshi, 2005; Oey et al., 2022). Over- or under-interpreting other agents within this cognitive hierarchy can be understood as both credulity and paranoia, important for different aspects of mental disorders (Alon, Schulz, Dayan, & Barnby, 2023; Barnby et al., 2023). It would be interesting to consider the effects of such recursive modeling in our task.

Point 3

Reviewer: *Technical point: 1. Was confidence ratings distribution similar between conditions? The random case seems to be most vulnerable to participants giving mostly low confidence ratings. Did this affect model fit/results?*

This is an interesting question.

In response, we extended the analysis beginning in Line 734:

> We provide further insights into the collected dataset by testing for differences in the confidence ratings distribution *(i)* between the experiments (full feedback and noisy conditions); *(ii)* within the experiments (fixing the feedback condition, but varying source types); and *(iii)* for differences within an experiment and between different source types (interaction).
> For *(i)*, we pooled confidence ratings across different source types for each condition and performed a permutation test to check whether the two conditions differed in their mean confidence rating. We used permutation tests because the distributions of confidence ratings were not Gaussian. The sample sizes per group were $N_1 = 28224$ confidence reports for the full feedback condition, and $N_2 = 14784$ confidence reports for the noisy feedback condition. While the difference in means was statistically significant ($|\mu_1 - \mu_2| = 2.51$ with $p \ll 0.05$) the effect size was small (Cohen's d 0.17). [1] This means that people trusted the sources more overall in the experiment that included full feedback.

> For *(ii)*, we pooled confidence ratings across the two different main conditions. We again used permutation tests to check for significant differences, but now between different source types. We, again, found statistically significant differences between all of the four source types. The pooled sample sizes were $N = 10752$ for each condition. We report the full values in Table 1. The strongest effect was between the helpful and the random source. The weakest was between helpful and blue-bias. This exactly mirrors the general findings of the main study.
> The analysis of interaction effects *(iii)* reveals that the largest effect (in both experiments) occurred between the helpful and random source. Notably, the findings in the noisy condition without feedback were particularly interesting. In contrast to the full feedback condition, there was nearly no effect between the helpful and blue-bias source, as well as between the opposite and random source. However, the differences of helpful and blue-bias showed a moderate effect when compared to the opposite and random conditions (magnitude of Cohens' d > 0.25, c.f. Table 1).

Table 1: Cohen's d values between different source types. We report the value pooled across full feedback and noisy, as well as the individual values (full feedback, noisy).

$\mu_1 \setminus \mu_2$	**Helpful**	**Random**	**Opposite**	**Blue-bias**
**Helpful**		0.52 (0.74, 0.4)	0.34 (0.32, 0.35)	0.13 (0.2, 0.08)
**Random**			-0.16 (-0.38, -0.03)	-0.38 (-0.52, 0.3)
**Opposite**				-0.21 (-0.12, -0.26)
**Blue-bias**				

Point 4

> **Reviewer:** *2. Line 260 – model fitting – some link to the model fitting procedure could be useful, as well as summary statistics about the parameter estimations (mean and range, for example) and indication of goodness of fit.*

Thank you for the thoughtful suggestion. In response, we have added summary statistics, including the mean and standard deviations of the fitted parameters, now shown in Table 3. For the beta priors, we report distributions in Figure 4 (full feedback) and Figure 8 (noisy).

Figure 3 and Figure 9 provide visual assessments such as the regression slopes and intercepts of the fit, and graphically illustrate the model's performance within the context of our study. Please note that our primacy focus was to highlight behavior under the various different conditions, rather than to engage in a detailed, quantitative model comparison – this would certainly be interesting, but is a bit beyond the scope of the paper.

We add to the supplementary material, beginning at Line 876: Further, Table 3 shows statistics of the fitted parameters.

Table 3: Fitted parameter statistics for the Bayesian model.

Parameter	Mean (ffb)	Std (ffb)	Mean (Noisy)	Std (Noisy)
beta priors blue (Fig. 4, Fig. 8)	0.56	0.13	0.66	0.18
beta priors green	0.63	0.12	0.70	0.14
κ	3.83	3.28	1.58	2.55
ω_α	1.08	0.47	n.a.	n.a.
ω_β	1.06	0.42	n.a.	n.a.
λ	0.04	0.11	n.a.	n.a.
ν	n.a.	n.a.	0.25	0.30
α_{forget}	n.a.	n.a.	28.33	13.44
β_{forget}	n.a.	n.a.	19.82	11.65

Point 5

> **Reviewer:** *3. Line 276 – binned analysis – I wonder whether using mixed effect logistic regression, with individuals' bias and slope as random variables, may not be a better way to study both individual level and group effects, instead of pulling ratings across participants and addressing individual slopes in two separate analyses.*

Thanks for the suggestion. We duly extended the analysis and fitted a mixed effects logistic regression model on participant data. While the mixed effects logistic regression provided a better BIC in general, yet we used the quarterly analysis in the main text to visualize the learning progress over time. The extended text in the supplementary starting at line 957:

> For further validation, we fit mixed effects Bayesian logistic regression. We constructed the model with group level slope and intercept parameters, as well as participant level slopes for the logistic regressors. For fitting, we used the glmmTMB package[2] in R. Including a participant level intercept was not significant. Although the mixed effects model achieved a better BIC score, we chose the quarter-based analysis for its ability to better illustrate learning behavior over the experiment. Our focus is not on exhaustive model comparison but on presenting an approach that most effectively conveys the behavioral dynamics.
> Most importantly, the mixed effects model agreed with our main findings using the quarter-based and half-based analyses of learning. That is, the group level slopes were significant for both conditions for all four source types and had the same signs as in the quarter-based analysis. The blue-bias source had a significant intercept in the full feedback condition, but not in the noisy condition. We report all parameter values in Table 5.

Table 5: Group-level parameters of mixed effects logisic regression on the participants learning trajectories. The stars indicate statistical significance against 5%.

Condition	Source Type	Slope	Intercept
full feedback	helpful	1.95*	-0.02
full feedback	random	0.54*	-0.01
full feedback	opposite	-0.66*	-0.01
full feedback	blue-bias	1.31*	-0.23*
noisy	helpful	1.28*	0.05
noisy	random	1.04*	0.01
noisy	opposite	0.25*	-0.03
noisy	blue-bias	1.17*	-0.05

Point 6

Reviewer: *4. Fig 9. It was very hard to see the empirical mean in panel B – lines are very similar to background.*

Sorry – we have now adjusted the figure for better visibility of the means, c.f. Figure 9.

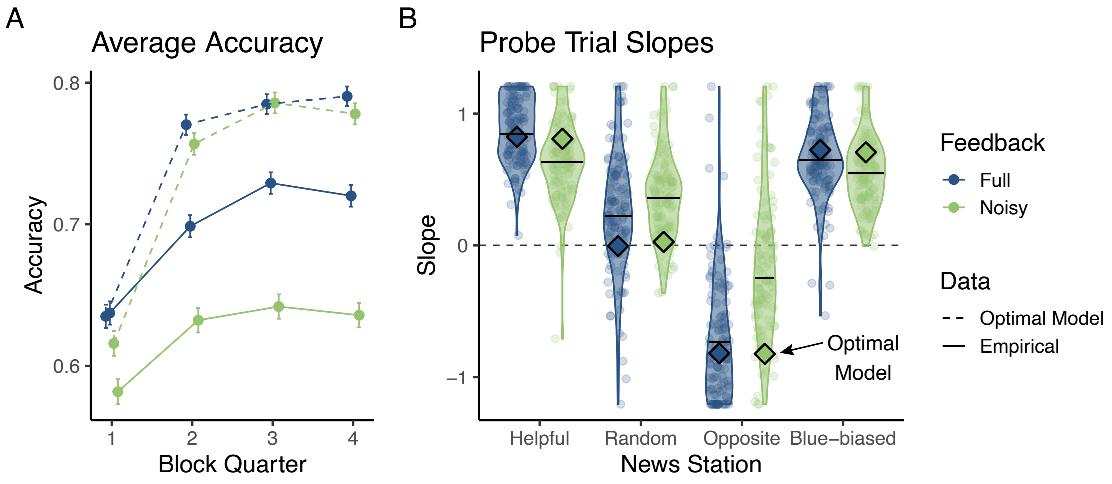

Figure 9: New Graphics with solid lines for means in Panel B.

Point 7

Reviewer: *5. Line 490 – slope and trust/intercept – there seem to be missing statistics in the text. Also, is the linear relation significant, or a mean value better describe these relations?*

Sorry – the statistics were reported in the appendix, in Table 1. We further added a statement about the statistical significance (Line 950):

> Slopes and intercepts of the winning models all reached statistical significance ($p < 0.05$).

Reviewer: *6. Line 550 - adaptively is written twice.*

Thanks for pointing this out - this has now been fixed.

Responses to Reviewer 3

Evaluation

> **Reviewer:** *This paper presents a new learning paradigm in which participants must infer the underlying state (blue/green) based on a signal as well as the reliability of the source of the signal (the news agency). Two experiments are reported. In the first experiment, participants receive unambiguous feedback on each trial about the true value (blue/green). In the second experiment, they receive ambiguous feedback in the form of several unbiased samples from an "independent council". Four kinds of news stations are tested: helpful, randomm, opposite, and blue-biased. Participant behavior is analyzed with a Bayesian model that also includes additional parameters (totling 9 parameters). The main result is that people in the first experiment are better at learning the news station mapping than in the second experiment. Additionally, people appear to perform much worse than optimal Bayesian.*
> *The paper tackles an interesting question and introduces a simple paradigm for understanding how people jointly reason about content and reliability of an information source. However, the main results seemed unsurprising, and even though there are interesting model-based analyses of the data, the paper would be improved if they were used in more of a way to advance an argument or investigate the underlying mechanisms at play. Some more specific points:*

We thank the reviewer for their feedback on our paper and appreciate their comments. We address them in full below.

Point 1 + 2

> **Reviewer:** *When introducing the model in the main text, the authors give very little intuition for what the model is supposed to capture other than that its Bayesian plus some other biases needed to fit the data. Why are these biases needed to capture the data? What happens if you fit the models without those biases? Some discussion of the qualitative features of the models with/without the biases is needed since one of the main takeaways from both experiments is that people *are not* Bayesian, even though the paper introduces the Bayesian solution as the working hypothesis for what people would be doing in the task.*

Thank you for the insightful feedback. We appreciate the opportunity to clarify the role of the biases and the broader framing of our model.

In particular, our goal was to frame the behaviour of participants within a Bayesian framework, albeit with approximations based on simple heuristic biases that are well established in the literature (see e.g. Benjamin, 2019): *(i)* non-linear transformation of confidence ratings, which reflects distortions in subjective confidence reports; *(ii)* an optimism/pessimism bias to mimic over- or underconfidence in belief updates; *(iii)* a forgetting factor, to account for recency effects in learning and imperfect memory integration. We think of these biases as part of the backdrop of our analysis, and so detailed them in the supplement. We were mainly seeking theoretical insights into the nature of inference, rather than the sort of detailed model comparison that would have been necessary if, for instance, we sought to use regressors from the model for matching with fMRI signals.

However, in the light of the reviewer's concerns here and below, we do now report a comparison with learning according to a simple Rescorla-Wagner rule, which has relevant structural differences from our approximate Bayesian treatment (see the next point).

Point 2 + 3

> **Reviewer:** *Building on the previous point, the authors should fit the data with different lesioned versions of the model and report the relative fits. That will give us a better sense of the work that each component of the model is doing and aid in interpreting the data.*

> **Reviewer:** *Its possible that people are not just Bayesian plus some biases, but using relatively simple heuristics or learning rules to solve the task. It would be good if the authors also tested some simple decision procedures used in the multi-armed bandit literature (since this task could be seen as a bandit with a slightly more abstract structure than usual). For example, could win-stay/lose-shift over trust/not-trust a news source be a strategy? What about a simple (non-Bayesian) reinforcement learning model like Rescorla-Wagner? One of the virtues of using such a simple task to study how people reason about potential sources of misinformation is that a more comprehensive model comparision is possible!*

Thank you very much for the suggestion. It is an excellent idea to compare the Bayesian model against a structurally simpler model.

We now include this analysis in the supplementary material, and discuss it in line 970 of the paper:

> In addition, we compare the Bayesian model against a structurally simpler model - a Rescorla-Wagner (RW) rule, since this operates according to related, but less sophisticated, incremental learning principles. The main difference between RW and Bayesian learning is the fixed learning rate in RW. Learning in the Bayesian model is fast initially (when epistemic uncertainty dominates) and slow ultimately (when aleatoric uncertainty dominates). Thus, if the participants are more Bayesian in their adaptation, then we can expect the RW to fit poorly, but in a way that might be hard to predict.
> For the RW, to allow for suitable generalization, we model Q values of observation x using a quadratic form as $Q(x) = w_0 + w_1 x + w_2 x^2$ and apply the logistic function to transform those Q values to probabilities. We found that a cubic term afforded no extra predictive power, but it was not practical to test the many other possible functional forms. Nevertheless, to provide RW with as much flexibility as possible, we allowed separate learning rates $\lambda_0, \lambda_1, \lambda_2$ for each parameter, per participant. Figure 17 shows fitted learning rates for those RW fits. To compare the learning behavior of the RW model together with the Bayesian model and our participants, we conducted an analysis which parallels that in the main paper: We simulated learning trajectories of the computational models, having fit any parameters to the behavior of the subjects. We then split the simulated trajectories and the behavior of the participants into two halves (akin to our procedure in the main part of the paper) and fit psychometric slopes and intercepts using Bayesian logistic regression as before. For each source type in each condition we used an ANOVA test to assess whether the slopes and intercepts were significantly different between the models and the data[3]. We found that the RW model had significantly lower slopes for nearly all source types and block halves in the full feedback condition (Figure 15). This suggests that the RW model lags behind the participants' (and the Bayesian model's) learning speed, likely due to its fixed learning rate. For the intercept, we only found a significant difference in the second block half in the blue-bias condition. The RW model's lower intercept compared to the participants (and the Bayesian model) suggests an overestimation.

We report the findings for the noisy condition in Figure 16. The results for the fitted intercepts agree with the full feedback version. The results for the slopes are more challenging to interpret, likely due to the additional noise. The slopes were lower for the RW than the participants in many conditions, albeit higher for the helpful and blue-bias source in the second half. Nevertheless, even when the ANOVA test implied significant differences across the three learning curves, the Bayesian model only significantly deviated from that of the participants in the first block of the helpful source.

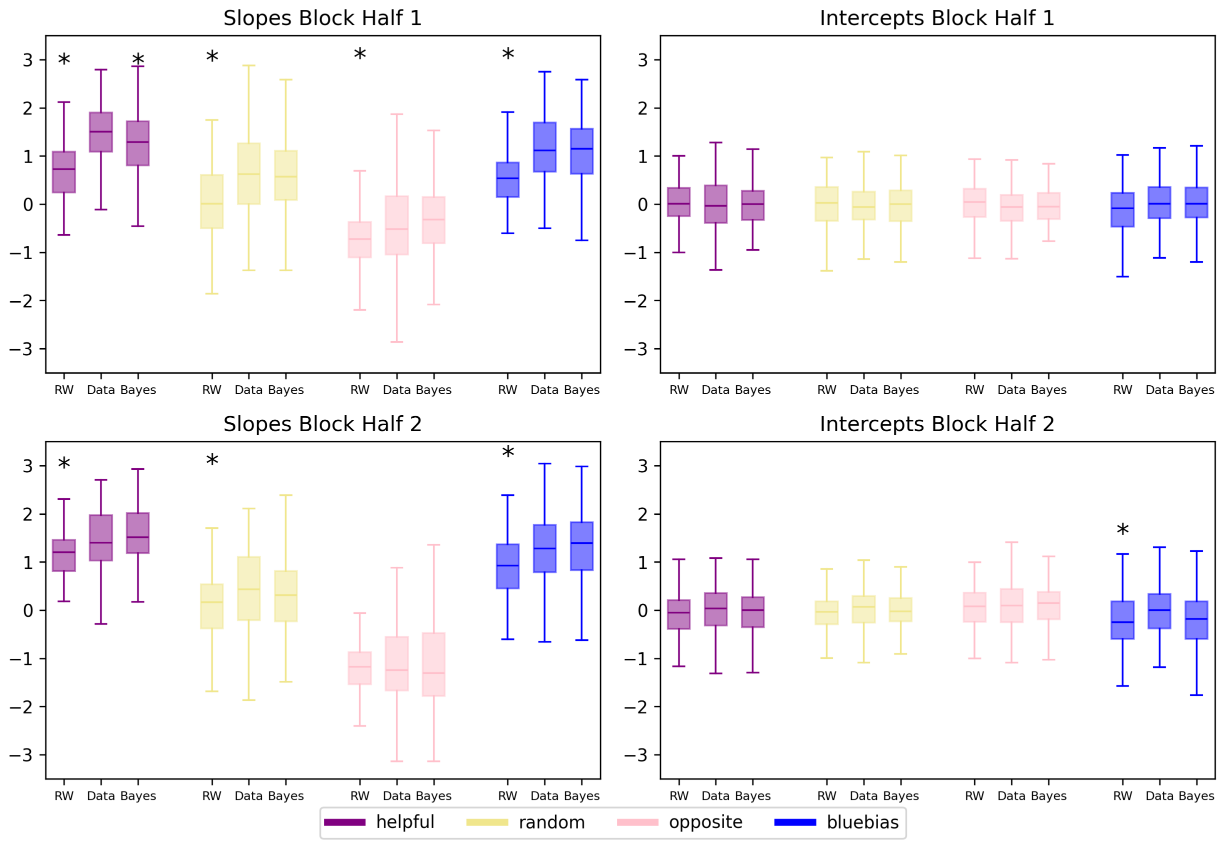

Figure 15: Model comparison in the full feedback condition. The asterisk indicates a significant ANOVA test for the source type, and marks whether the respective model deviates from the participant data according to a t-test.

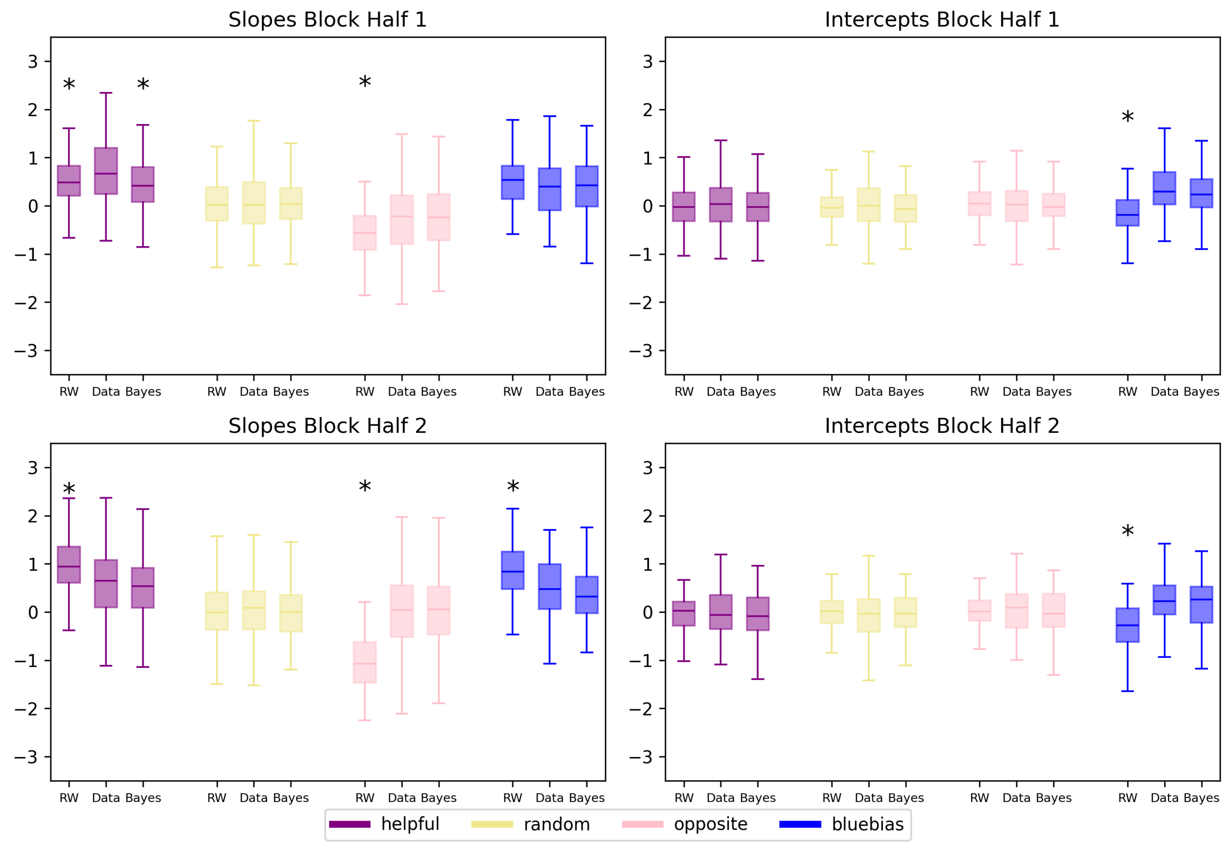

Figure 16: Model comparison in the noisy condition. The asterisk indicates a significant ANOVA test for the source type, and marks whether the respective model deviates from the participant data according to a t-test.

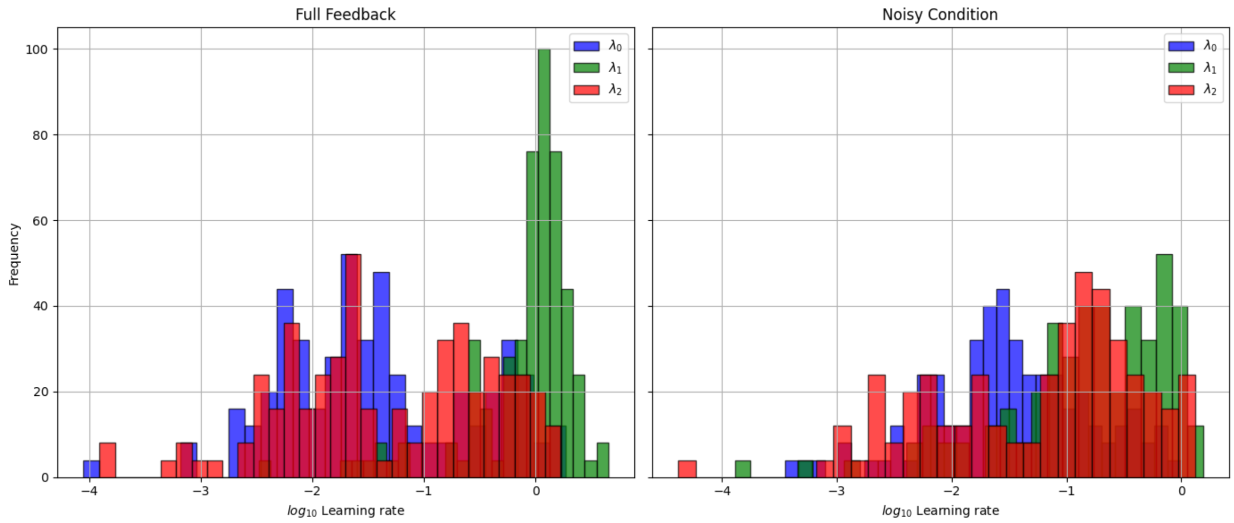

Figure 17: Fitted earning rates of the Rescorla Wagner Model

Point 4

Reviewer: *4. It wasn't clear to me from the main text that people were incentivized to learn about the truth.*

The main text (Line 143) now includes

> 'They [the participants] were incentivized to make the correct choice and report their confidence faithfully.'

We provide more details in the supplementary material, when we provide detail on the participants and data collection. This paragraph now says (Line 729)

> Participants were remunerated with a base payment and a performance based bonus computed via the quadratic scoring rule based on their confidence judgements during the main block.

The quadratic scoring rule has been shown to be a good way to incentivize correct probabilistic estimates about noisy states.

Point 5

Reviewer: *5. "To answer this, we next analyzed participants' learning trajectories over the first 28 trials of a block." - does this just mean you're not analyzing the probe trials? It would be clearer if this was stated directly maybe by referring to these as the "main trials".*

Sorry for this ambiguity. We edited the text (Line 322) as:

> Our analysis of the probe trials showed that participants were generally able to learn the statistical regularities of these sources - but how did they arrive at these conclusions? To answer this, we next analyzed participants' learning trajectories over the first 28 trials of a block (excluding the probe trials).

Point 6

Reviewer: *6. "demarcated probe trials" - were these demarcated to the participants?*

Yes, the probe trials were demarcated to the participants - they were shown after a short break. Line 259:

> To test participants' beliefs in the sources in a standardized manner, each block ended with an additional six ~~demarcated probe trials~~ probe trials, clearly demarcated by a short break, in which participants saw all possible constellations of blue and green endorsements $X_{I,t} \in \{0, 1, 2, 3, 4, 5\}$ in random order.

Point 7

Reviewer: *7. I couldn't find an explicit statement about how many blocks of trials were in each experiment.*

Sorry - we have edited the text (Line 257) as follows:

> [...] each of the sources for a total of 28 trials. We presented the sources in a blocked manner, counterbalancing the order between participants. Thus, the whole experiment consisted of 4 blocks, each comprising 28 learning trials plus 6 probe trials.

Point 8

> **Reviewer:** *8. " Participants played with each of the sources for a total of 28 trials. " - "played" was a peculiar choice of word and I wasn't sure if it just meant they received 28 trials for that source or interacted with the interface in a more extended way.*

Sorry for the unfortunate wording. Line 255 now reads:

> Participants ~~played~~ interacted with each of the sources for a total of 28 learning trials. We presented the sources in a blocked manner, counterbalancing the order between participants.

References

Alon, N., Schulz, L., Dayan, P., & Barnby, J. M. (2023). Between prudence and paranoia: Theory of Mind gone right, and wrong. *ICML 2023 Workshop on Theory of Mind in Communicating Agents*.

Alon, N., Schulz, L., Rosenschein, J. S., & Dayan, P. (2023). A (Dis-)information Theory of Revealed and Unrevealed Preferences: Emerging Deception and Skepticism via Theory of Mind. *Open Mind*, *7*, 608–624. https://doi.org/10.1162/opmi_a_00097

Asch, S. (1951). Effects of group preassure the modification and distortion of judgment. *Groups, leadership and men*.

Austen-Smith, D., & Banks, J. S. (1996). Information aggregation, rationality, and the condorcet jury theorem. *American political science review*, *90*(1), 34–45.

Barnby, J. M., Dayan, P., & Bell, V. (2023). Formalising social representation to explain psychiatric symptoms. *Trends in Cognitive Sciences*, *27*(3), 317–332. https://doi.org/10.1016/j.tics.2022.12.004

Benjamin, D. (2019). Chapter 2-errors in probabilistic reasoning and judgment biases. volume 2 of handbook of behavioral economics: Applications and foundations 1. *need to cite this properly*.

Bhui, R., & Gershman, S. J. (2020). Paradoxical effects of persuasive messages. *Decision*, *7*(4), 239–258. https://doi.org/10.1037/dec0000123

Breakstone, J., Smith, M., Connors, P., Ortega, T., Kerr, D., & Wineburg, S. (2021). Lateral reading: College students learn to critically evaluate internet sources in an online course. *Harvard Kennedy School Misinformation Review*. https://doi.org/10.37016/mr-2020-56

Celadin, T., Capraro, V., Pennycook, G., & Rand, D. G. (2023). Displaying News Source Trustworthiness Ratings Reduces Sharing Intentions for False News Posts. *Journal of Online Trust and Safety*, *1*(5). https://doi.org/10.54501/jots.v1i5.100

Dias, N., Pennycook, G., & Rand, D. G. (2020). Emphasizing publishers does not effectively reduce susceptibility to misinformation on social media. *Harvard Kennedy School Misinformation Review*. https://doi.org/10.37016/mr-2020-001

Farrell, J., & Matthew, R. (1996). Cheap Talk. *Journal of Economic Perspectives*, *10*(3), 103–118.

Gmytrasiewicz, P. J., & Doshi, P. J. (2005). A framework for optimal sequential planning in multiagent settings. *Journal of Artifical Intelligence Research*, *24*, 49–79.

Hertwig, R., & Engel, C. (2021, March). *Deliberate Ignorance: Choosing Not to Know*. MIT Press.

Jara-Ettinger, J., Gweon, H., Schulz, L. E., & Tenenbaum, J. B. (2016). The Naïve Utility Calculus: Computational Principles Underlying Commonsense Psychology. *Trends in Cognitive Sciences*, *20*(8), 589–604. https://doi.org/10.1016/j.tics.2016.05.011

Lindström, B., Jangard, S., Selbing, I., & Olsson, A. (2018). The role of a "common is moral" heuristic in the stability and change of moral norms. *Journal of Experimental Psychology: General*, *147*(2), 228.

Nitzan, S. (2009). *Collective preference and choice*. Cambridge University Press.

Oey, L. A., Schachner, A., & Vul, E. (2022). Designing and Detecting Lies by Reasoning About Other Agents. *Journal of Experimental Psychology: General*. https://doi.org/10.1037/xge0001277

Pennycook, G., & Rand, D. G. (2019). Fighting misinformation on social media using crowdsourced judgments of news source quality. *Proceedings of the National Academy of Sciences*, *116*(7), 2521–2526. https://doi.org/10.1073/pnas.1806781116
